# 本科生综合实验: 4-[二(4-甲基苯)氨基]苯甲醛合成与变色性能研究

**DOI:** 10.3724/SP.J.1123.2023.11009

**Published:** 2024-03-08

**Authors:** Yuzhen PAN, Han XUE, Yalin GAO, Shurui HU, Wenzhu ZHANG

**Affiliations:** 大连理工大学化学学院, 辽宁 大连 116024; School of Chemistry, Dalian University of Technology, Dalian 116024, China

**Keywords:** 综合性实验, 三苯胺类化合物, 溶剂化变色, 机械致荧光变色, comprehensive experiment, triphenylamine compounds, solvatofluorochromism, mechanoflurochromism

## Abstract

针对有机合成实验缺少性质研究、仪器分析实验分析方法相对独立的问题,本项目基于机械致荧光变色材料在化学前沿领域的研究,设计了一个本科生综合实验。以4,4'-二甲基三苯胺为原料,通过Vilsmeier-Haack反应合成了4-[二(4-甲基苯)氨基]苯甲醛,并通过质谱、红外吸收光谱和核磁共振波谱对其结构进行了表征;然后通过紫外-可见吸收光谱、荧光光谱等对其溶剂化变色和机械致荧光变色现象进行了研究,并通过X射线粉末衍射对其机械致荧光变色机理进行了探讨。本项目将有机合成与仪器分析实验相结合,形成合成及变色性能一体化研究,实验综合性强,现象有趣,是一个兼具趣味性、科学性和综合性的本科生创新实验。该实验可以很好地激发学生对化学研究的兴趣,锻炼多项实验操作能力,提升学生的综合科研素质。

## Abstract (en)

To solve the problems of the lack of property research in organic synthesis experiments and the relative independence of instrumental analytical methods in experiments, we designed a comprehensive undergraduate experiment based on mechanofluorochromic materials. In this project, 4-[bis(4-methylphenyl)amino] benzaldehyde was synthesized via the Vilsmeier-Haack reaction using 4,4'-dimethyltriphenylamine as the raw material. The product was then characterized by mass spectrometry, infrared absorption spectroscopy, and nuclear magnetic resonance spectroscopy. The solvatofluorochromism and mechanofluorochromism of the target material were studied using ultraviolet-visible absorption spectroscopy, fluorescence spectroscopy, etc. Furthermore, the mechanism of mechanofluorochromism was determined using powder X-ray diffraction. Organic synthesis and a series of instrumental analytical methods were combined to form an integrated experiment. The experiment is interesting, scientific, and comprehensive for undergraduates as a creative exercise; moreover, it can inspire their interest in chemical research, cultivate a variety of experimental operation abilities, improve creative-thinking skills, and encourage the development of effective solutions to existing problems in chemical experiments.

综合实验对学生综合素质、创新能力及团队协作能力的培养至关重要。在现有的实验教学中,多数实验课程相对独立,比如有机实验一般侧重于有机化合物的合成,但对化合物性质研究涉及较少;而仪器分析实验大多是单个实验独立进行,各个仪器分析方法之间的关联性较弱,缺乏对学生整体实验能力的培养,不利于学生创新思维能力的提升。随着化学学科的飞速发展,各高校对综合实验教学改革进行了诸多有益的探索及实践,并融入学科前沿,让学生体会到学科交叉融合和前沿科学研究的魅力^[1,2]^。

机械致荧光变色材料是一类在研磨、挤压等机械应力的作用下,荧光颜色发生变化的材料,在传感器、数据存储、智能开关等领域具有广泛的应用前景^[3,4]^。目前机械致荧光变色材料有许多种类,包括含噻唑基、吩噻嗪基、吡啶基、咔唑基等基团的化合物,四苯乙烯类衍生物,三苯胺类衍生物和金属有机配合物等^[5,6]^。机械致荧光变色过程通常被认为是施加的机械应力改变了化合物分子的排列方式,该过程不会破坏化合物的化学结构,因此可通过溶剂熏蒸或退火使其恢复到初始状态^[7]^。

本实验以4,4'-二甲基三苯胺(DMTPA)为原料,通过Vilsmeier-Haack反应合成了一个具有机械致荧光变色性质的分子4-[二(4-甲基苯)氨基]苯甲醛(BMABA)^[8,9]^。通过质谱、红外吸收光谱、核磁共振波谱对产物进行了结构表征;使用紫外-可见吸收光谱、荧光光谱对其光物理性质进行了研究。目标分子在不同的溶剂中呈现明显的溶剂化变色现象,且在研磨、挤压等机械应力的作用下其固体荧光颜色由蓝光变为蓝绿色光,可视化的实验现象能够激发学生的探究兴趣。

本实验涉及有机合成反应机理、电子分布与能级跃迁等理论知识,可以有效加深学生对化学物质的结构-性质-功能关系的理解。实验涉及化合物制备、仪器分析表征、发光材料性能评价等科研实验过程中必需的技能,将有机合成实验与仪器分析实验相结合,形成了化合物合成及性质研究一体化实验。本实验兼具趣味性、科学性和综合性,可以提升学生综合运用多学科知识分析和解决实际问题的能力,激发学生的科研兴趣,为综合实验项目的设计提供参考。

## 1 实验部分

### 1.1 实验原理

本实验有机合成部分采用的反应是Vilsmeier-Haack反应,即由*N*,*N*-二甲基甲酰胺(DMF)和三氯氧磷(POCl_3_)作用生成一种氯代亚胺盐(Vilsmeier试剂),与富电子的芳香族化合物通过芳香亲电取代发生甲酰化反应,生成醛类化合物,其合成路线及反应机理见图1。

**图 1:**
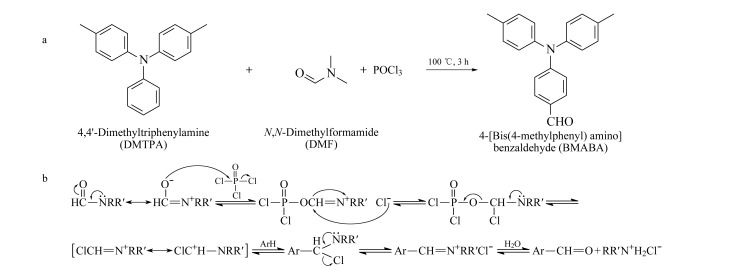
(a)4-[二(4-甲基苯)氨基]苯甲醛的合成路线与(b)Vilsmeier-Haack反应机理图

### 1.2 仪器、试剂与材料

Q-TOF Micro质谱仪(沃特世科技有限公司,美国); Nicolet 6700红外吸收光谱仪(赛默飞世尔科技公司,美国); Ascend^TM^ 400M核磁共振波谱仪(布鲁克科技有限公司,德国); Cary 300紫外-可见吸收光谱仪(安捷伦科技有限公司,美国); FL6500荧光光谱仪(珀金埃尔默仪器有限公司,美国); X' Pert PRO X射线粉末衍射仪(马尔文帕纳科公司,荷兰)。

DMTPA(98%,上海阿拉丁生化科技股份有限公司); DMF(分析纯,天津市大茂化学厂);POCl_3_(分析纯,萨恩化学技术(上海)有限公司);碳酸氢钠、正己烷、二氯甲烷、三氯甲烷、四氢呋喃、乙酸乙酯、丙酮、甲醇均为分析纯试剂(天津市大茂化学厂)。

### 1.3 目标化合物的合成、表征及性质测试

#### 1.3.1 合成步骤

将DMTPA(1.37 g, 5.01 mmol)溶于DMF(2.3 mL, 29.8 mmol)中,在冰水浴条件下缓慢加入三氯氧磷(0.46 mL, 5.02 mmol)。加入完毕后,调节反应温度为100 ℃,加热3 h。冷却至室温后,将产物溶于二氯甲烷,然后用碳酸氢钠溶液中和,萃取,经无水Na_2_SO_4_干燥后,通过减压蒸馏收集固体产物,以二氯甲烷和甲醇(*V*_二氯甲烷_:*V*_甲醇_=100:1)为展开剂,用硅胶柱进行色谱分离,得到淡黄色BMABA(1.14 g,产率75.6%)^[8,9]^。

#### 1.3.2 结构表征

用质谱仪、红外吸收光谱仪和核磁共振波谱仪对产物的结构进行分析。

#### 1.3.3 性质测试

溶剂化变色:准确称量BMABA,分别以二氯甲烷、三氯甲烷、丙酮、正己烷、四氢呋喃、乙酸乙酯为溶剂配制成浓度为1.00×10^-5^ mol/L的试样,用于紫外-可见吸收光谱仪和荧光光谱仪分析。

机械致荧光变色:取少量BMABA均匀放入玛瑙研钵中摩擦,在紫外灯照射下观察其机械致荧光变色现象,然后向摩擦后的固体中滴加少量二氯甲烷,待固体溶解后,静置,待二氯甲烷挥发后得到固体产物(注:此步骤必须在通风橱中操作)。分别测定BMABA摩擦前、摩擦后及经二氯甲烷处理后的荧光光谱图,并用X射线粉末衍射仪测定其摩擦前后的晶体结构变化。

## 2 结果与讨论

### 2.1 质谱分析

已知BMABA相对分子质量为301.38,其质谱图中分子离子峰[M+H]^+^的*m/z*为302.18,如图2所示,与BMABA的相对分子质量基本吻合,表明合成产物即为目标化合物。

**图 2:**
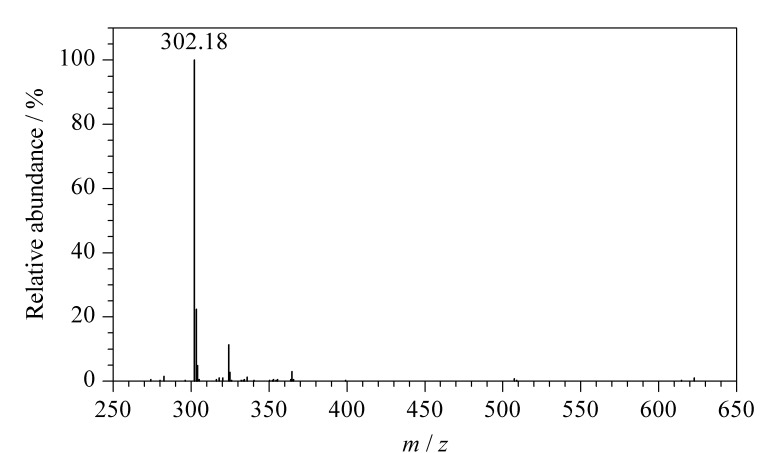
BMABA的质谱图

### 2.2 红外吸收光谱分析

由图3可知,与DMTPA的红外吸收光谱相比,BMABA的红外吸收光谱在1679.81 cm^-1^处有一个明显的羰基吸收峰,归属为BMABA的醛基振动吸收峰,由于该醛基与苯环共轭,羰基峰红移,与BMABA结构吻合,红外吸收光谱分析结果证明已合成出目标产物,与质谱分析结果一致。

**图 3:**
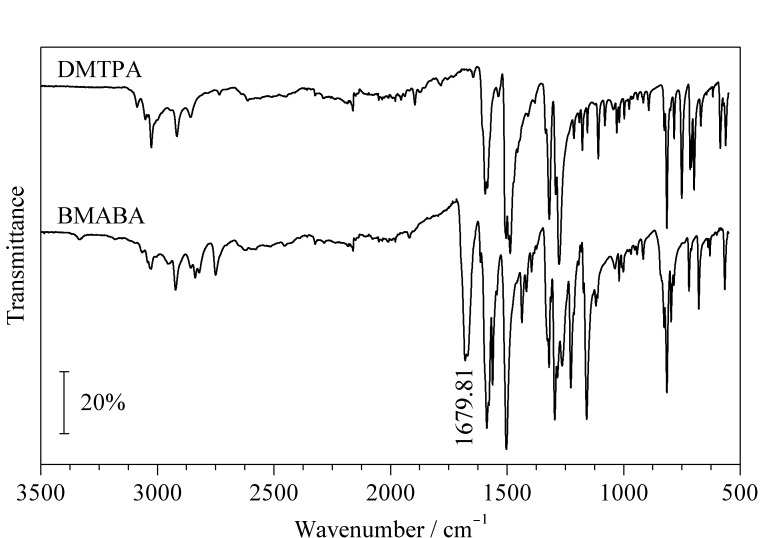
DMTPA和BMABA的红外吸收光谱图

### 2.3 核磁共振波谱分析

BMABA的核磁波谱分析结果如图4所示,^1^H NMR (400 MHz, CDCl_3_): *δ*_H_ (ppm)=2.32 (s, 6H), 6.91~7.15 (d, *J*=8.5 Hz, 2H), 7.20~7.37 (d, *J*=8.5 Hz, 8H), 7.60~7.70 (d, *J*=8.5 Hz, 2H), 9.89 (s, 1H),与BMABA结构吻合^[10,11]^。化学位移为2.32的单峰是甲基上的氢,7~8的多重峰是苯环上的氢,9~10的单峰是醛基上的氢。这证明已合成出目标产物,与质谱及红外光谱分析结果一致。

**图 4:**
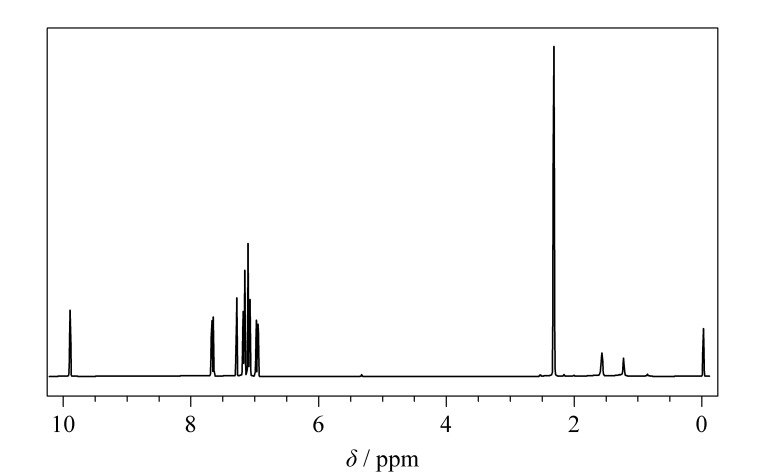
BMABA的氢核磁共振波谱图

### 2.4 溶剂化变色

如图5a所示,在可见光下,样品在不同溶剂中颜色基本为无色,无明显差异;如图5b所示,在紫外灯365 nm照射下颜色差异较大,呈现明显的溶剂化变色现象。

**图 5:**
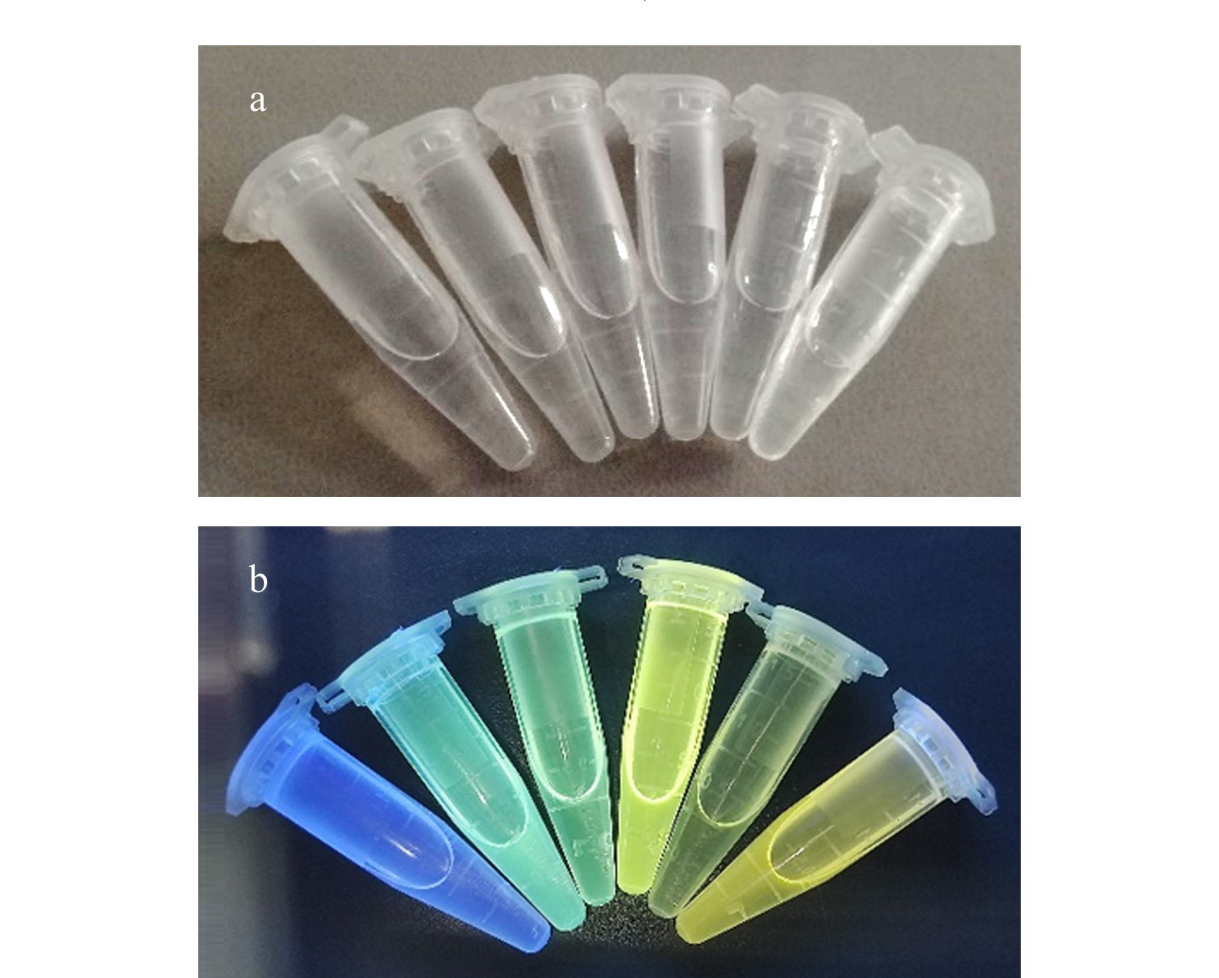
BMABA在(a)可见光和(b)紫外光(365 nm)照射下在不同溶剂中的照片图

**图 6:**
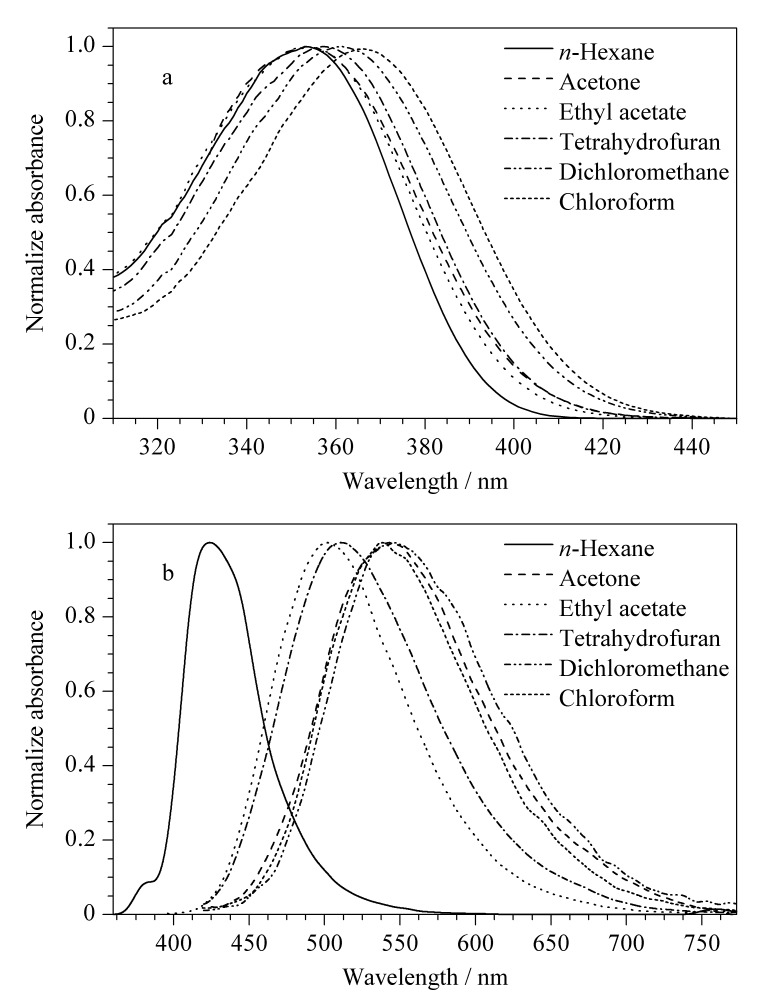
BMABA在不同溶剂中的(a)紫外吸收光谱图和(b)荧光发射光谱图

将配制好的一系列浓度为1.00×10^-5^ mol/L的试样进行紫外吸收光谱分析和荧光光谱分析,结果见图6。由图6a可以看出,由于BMABA的羰基和苯环发生共轭,电子发生*π*→π^*^跃迁产生K吸收带,极性溶剂使K带发生红移;随着溶剂极性的增加,其紫外吸收光谱最大吸收峰红移。由图5和图6b可以看出,BMABA在正己烷中呈蓝紫色,对应波长424 nm;在乙酸乙酯和四氢呋喃中呈绿色,分别对应波长502 nm和507 nm;在三氯甲烷和丙酮中为黄色,分别对应波长538 nm和542 nm;在二氯甲烷中为橙黄色,对应波长546 nm, BMABA在不同溶剂中紫外光下呈现的颜色与其荧光发射光谱图的最大发射峰相吻合。这是因为分子内电子发生了*π*→π^*^跃迁,在激发态时分子内的电荷转移程度较大,并且醛基的存在使得分子内电荷向两极分布程度加剧。由于激发态的弛豫分子结构与基态的弛豫分子结构显著不同,化合物的构象也发生了改变,即发生了扭曲的分子内电荷转移(twisted intramolecular charge transfer, TICT)。随着溶剂极性的增大,对激发态比基态的稳定作用更大,荧光发射波长随着溶剂极性的增大而向长波长方向移动^[11⇓⇓⇓-15]^。

### 2.5 机械致荧光变色

BMABA具有机械致荧光变色性质。由图7可以看出,BMABA在受到机械外力作用后,在室温可见光照射下,颜色未发生明显变化;在365 nm紫外光照射下,颜色由蓝色变为蓝绿色,相应地,其荧光发射波长由471 nm红移至485 nm(其荧光发射光谱如图8)。

**图 7:**
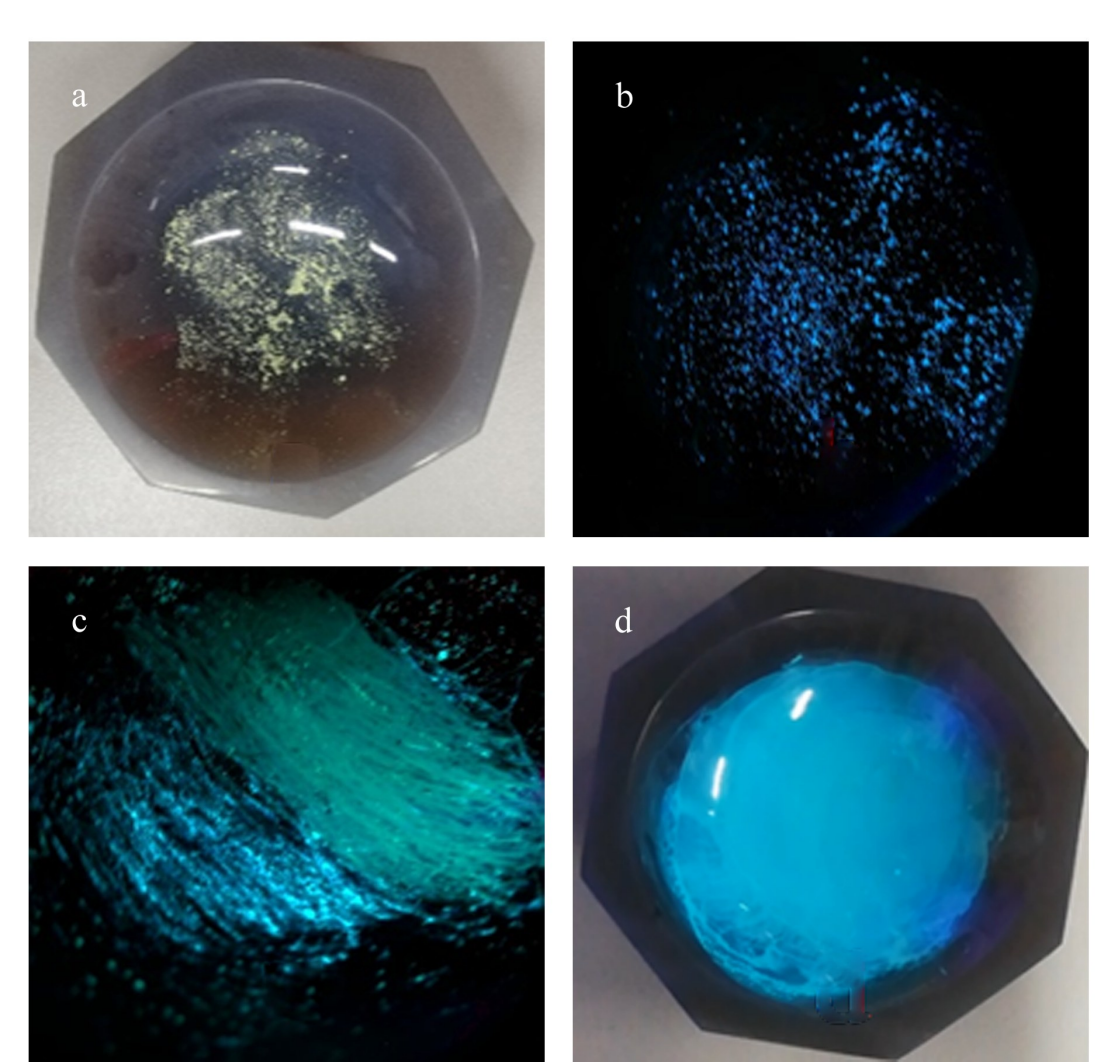
不同条件下的BMABA照片

**图 8:**
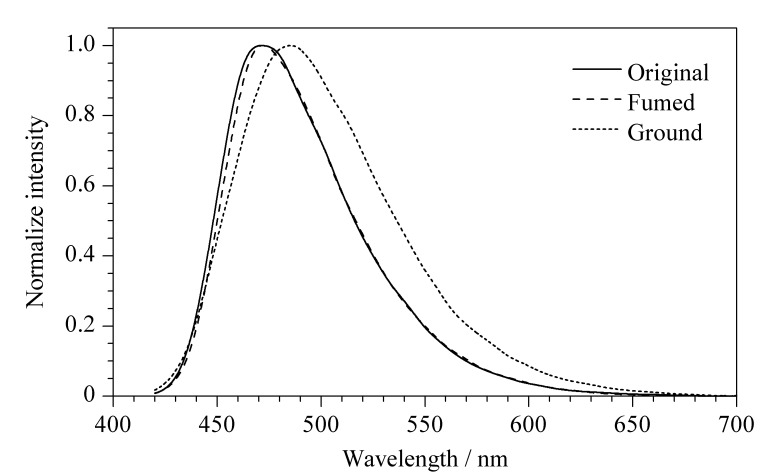
BMABA在初始、研磨、溶剂熏蒸状态下的荧光发射光谱图

研磨后的发光化合物经溶剂处理后可实现发光颜色的可逆性转变,且可逆性良好(见图7d和图8)。这是由于摩擦后,BMABA由晶态转变为无定形态。在晶态时BMABA的分子排列紧密,处于激发态时无法形成有效的TICT结构,而其在非晶态时分子间的自由体积较大,能够形成TICT结构,使得激发态分子更加稳定,荧光波长红移^[11⇓⇓⇓-15]^。图9为BMABA在机械摩擦前后的X射线粉末衍射图,由于摩擦后样品的非晶态不是很稳定,再结晶速度较快,因此XRD谱图记录到摩擦后部分晶态变化。

**图 9:**
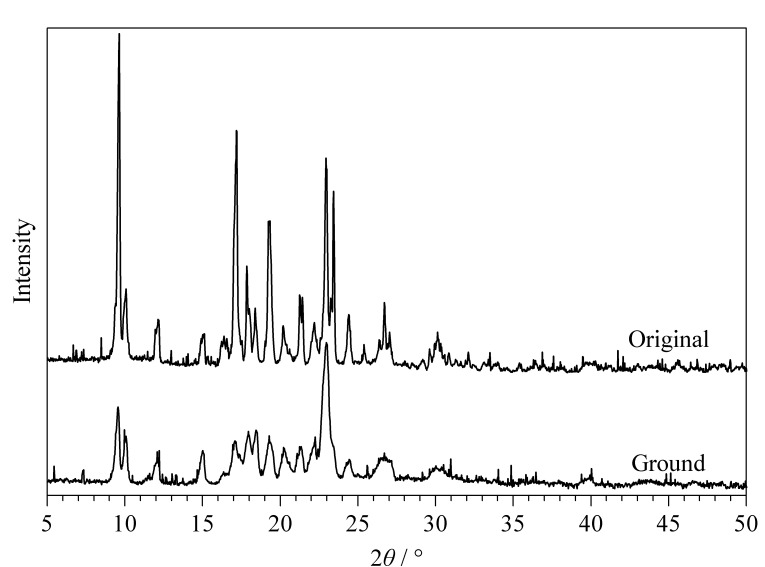
BMABA机械摩擦前后的X射线粉末衍射图

该变色材料的变色性能显著,其溶剂化变色可用于对环境溶剂进行准确的极性检测;而机械致荧光变色性质可用于智能感知或传感器等应用领域,如在物体表面涂覆该材料,通过其是否变色掌握物体变形信息和损伤程度等,以智能监测物体的实时状态。

## 3 实验的组织实施及教学反思

### 3.1 实验的组织实施及教学反馈

本实验安排为16学时,可分2次完成。第1次(8学时):目标化合物的合成及提纯(柱色谱分离);第2次(8学时):红外光谱分析、核磁共振波谱分析、溶剂化变色及机械致荧光变色性质研究、紫外-可见吸收光谱测试、荧光光谱测试、XRD粉末衍射表征、实验结果讨论与总结。如需进一步拓展实验内容,可结合X射线单晶衍射仪、综合物性测量仪对分子材料进行结构及热分析表征,该部分内容为2学时。

实验结束后,调研了学生对本实验的评价和收获,结果表明,学生对该综合创新实验表现出浓厚的兴趣。同学们表示:该合成与变色性能研究实验变色现象明显,具有趣味性和新颖性,而且对前沿文献的调研也拓宽了视野,综合分析表征技能得到了提升,自己的实验设计及创新思维得到了锻炼。这些反馈信息也表明,将学科前沿转化为实验教学对学生综合能力的提升大有裨益。

### 3.2 实验教学中存在的问题及注意事项等

(1)本实验有机合成模块反应时间较长,建议1人1组完成合成部分工作,并在等待的时间进行文献研讨或开放实验设计等教学活动,充分发挥学生主动性。(2)性质测试部分涉及多种大型仪器操作,建议2人一组分工、协作完成实验。(3)合成步骤中,所用的DMF和二氯甲烷试剂预先干燥处理。(4)POCl_3_为无色透明的发烟液体,有刺激性气味和腐蚀性,使用时应戴手套并在通风橱中进行操作,并在液体滴加完毕后及时将注射器放入碱性溶液中处理(POCl_3_遇水生成磷酸和盐酸)。(5)本实验所用的油浴温度达100 ℃,在反应过程中应防止被烫伤。(6)大型仪器操作应遵循仪器使用规则,如禁止携带铁磁性物体靠近磁体等。

## 4 结论

本实验通过Vilsmeier-Haack反应,采用4,4'-二甲基三苯胺在*N*,*N*-二甲基甲酰胺和三氯氧磷作用下合成了目标分子4-[二(4-甲基苯)氨基]苯甲醛,通过质谱、红外吸收光谱、核磁共振波谱对产物进行了结构表征,并用紫外可见吸收光谱、荧光光谱等分析方法对其溶剂化变色和机械致荧光变色性质进行了详细研究。

本实验操作简单,实验现象有趣,结构性质表征方法丰富且易于操作,可提高学生对于探究性实验的兴趣;有机合成实验与仪器分析实验均可独立实施,满足不同层次学生个性化科研能力培养需求,且两个模块可组合成一个综合性实验,完整呈现科学研究过程。本综合实验实现了合成、结构表征及变色性能一体化研究,极大地锻炼了学生的整体实验能力,为学生未来从事科研工作打下了坚实的基础。后续还可以4-[二(4-甲基苯)氨基]苯甲醛为起始物,设计新的分子结构并研究其性质,形成开放创新体系,有利于提升学生的综合创新能力,促进化学实验教学提质增效。
